# Application of a linear interpolation algorithm in radiation therapy dosimetry for 3D dose point acquisition

**DOI:** 10.1038/s41598-023-31562-3

**Published:** 2023-03-20

**Authors:** Yixiao Guo, Bo Li, Yazhou Li, Wen Du, Weigui Feng, Shifang Feng, Guoying Miao

**Affiliations:** 1 Department of Radiation Oncology, Gansu Provincial Hospital, Lanzhou, 730000 People’s Republic of China; 2 Department of Bone and Soft-Tissue Carcinoma, Gansu Provincial Hospital, Lanzhou, 730000 People’s Republic of China

**Keywords:** Biomedical engineering, Radiotherapy

## Abstract

Air-vented ion chambers are generally used in radiation therapy dosimetry to determine the absorbed radiation dose with superior precision. However, in ion chamber detector arrays, the number of array elements and their spacing do not provide sufficient spatial sampling, which can be overcome by interpolating measured data. Herein, we investigated the potential principle of the linear interpolation algorithm in volumetric dose reconstruction based on computed tomography images in the volumetric modulated arc therapy (VMAT) technique and evaluated how the ion chamber spacing and anatomical mass density affect the accuracy of interpolating new data points. Plane measurement doses on 83 VMAT treatment plans at different anatomical sites were acquired using Octavius 729, Octavius1500, and MatriXX ion chamber detector arrays, followed by the linear interpolation to reconstruct volumetric doses. Dosimetric differences in planning target volumes (PTVs) and organs at risk (OARs) between treatment planning system and reconstruction were evaluated by dose volume histogram metrics. The average percentage dose deviations in the mean dose (D_mean_) of PTVs reconstructed by 729 and 1500 arrays ranged from 4.7 to 7.3% and from 1.5 to 2.3%, while the maximum dose (D_max_) counterparts ranged from 2.3 to 5.5% and from 1.6 to 7.6%, respectively. The average percentage dose/volume deviations of mixed PTVs and OARs in the abdomen/gastric and pelvic sites were 7.6%, 3.5%, and 7.2%, while mediastinum and lung plans showed slightly larger values of 8.7%, 5.1%, and 8.9% for 729, 1500, and MatriXX detector arrays, respectively. Our findings indicated that the smaller the spacing between neighbouring detectors and the more ion chambers present, the smaller the error in interpolating new data points. Anatomical regions with small local mass density inhomogeneity were associated with superior dose reconstruction. Given a large mass density difference in the various human anatomical structures and the characteristics of the linear interpolation algorithm, we suggest that an alternative data interpolation method should be used in radiotherapy dosimetry.

## Introduction

For advanced radiation therapy techniques such as VMAT and RapidArc (Varian Medical System, a treatment planning and delivery method derived from VMAT) with higher delivery efficiency^1^, the radiation field in each control point is divided into multiple sub-fields with varying aperture sizes due to the movement of muti-leaf collimator leafs. During dose delivery, these sub-fields are changed over time to adjust the intensity distribution of the field^2,3^. To ensure that the delivered dose is consistent with the treatment planning system (TPS) dose within a clinically defined tolerance range^4^, pseudo-3D detector array geometries are commonly used to represent measurement geometries in clinical VMAT/RapidArc planning^4–6^, prior to the patient’s first treatment fraction. Generally, measured doses are compared to the TPS dose by calculating the γ pass rate and/or reconstructing dose based on patientsʼ anatomical structures on CT images^6^. Because detector arrays such as ion chambers and semiconductors can provide the unambiguous interpretation on measurement dose and the ability to compare measurement to calculation within clinically meaningful accuracy thresholds, a variety of studies and products have been developed for extracting dose distributions on anatomical structures and verifying external beam radiation treatment plans with 2D or 3D detector arrays^7–10^. However, these data points exist only in discrete individual detectors. Additionally, a detector arrayʼs dosimetric characteristics and spatial resolution will also vary. For instance, p-type silicon diodes used by Delta 4 (Scandidos, Uppsala, Sweden) are spaced at 0.5 cm intervals in the central 6 × 6 cm^2^ region, and 1 cm spacing outside the central area of the array^7^. The center spacing of adjacent ion chambers in the Octavius 729 detector array (PTW, Freiburg, Germany) is 1 cm, while the spacing of the Octavius 1500 detector array (PTW, Freiburg, Germany) is 0.707 cm^11^. For TPS dose calculation, different computational grids can be used. Intensity-modulated radiation therapy (IMRT) and VMAT plans may use a computational grid of 2–3 mm, while radiotherapy techniques such as stereotactic body radiotherapy and stereotactic radiosurgery may use a 1 mm grid^4^. Ideally, the delivered dose distribution should present a spatial resolution equal to or higher than the TPS dose distribution. Conversely, the delivered dose distribution should be interpolated to a finer grid size^4^. To overcome the defects of fewer sampling points associated with lower spatial resolution, a detector array is generally provided with an interpolation method^12^. Interpolation, namely, spatial data interpolation, is aimed at increasing the number of data points by estimating dose values in the neighbourhood of single detectors. The selection of different interpolation algorithms is the main factor affecting the interpolation accuracy^13–15^, with the linear interpolation algorithm being the most commonly used. Studies of the dose reconstruction have indicated that dosimetric systems mostly reconstruct volume doses using linear interpolation by resolving the measured dose to the TPS grid resolution, and the differences between reconstructed and calculated doses are evaluated by the γ pass rate method^8,14–19^. Taken together, as revealed by previous reports, linear interpolation has resulted in larger changes in the γ pass rate^12,16–18^.

Considering that the historical γ metric method cannot provide additional information about failure points^15^, reconstructed doses on CT images can achieve a more representative and comprehensive quality assurance on radiotherapy planning. A direct dosimetric comparison can be performed in regions of interest by the reconstructed and planned DVHs^4,5^. To date, however, how the linear interpolation affect the accuracy of the reconstructed 3D volume dose and subsequently the DVHs metrics calculation have not been evaluated, and it also remains unclear how does the linear interpolation algorithm affect the accuracy of dose reconstruction in human regions of interest with different mass densities and scattering abilities. Herein, by investigating the potential principle of the linear interpolation algorithm using plane measurement doses by Octavius and MatriXX (IBA, Schwarzenbruck, Germany) ion chamber detector arrays, we evaluated how different detector resolution and anatomical structures with various mass density ranges affect the accuracy of interpolating new data points in VMAT technique. Our results may contribute to a better understanding of the advantages and disadvantages of the linear interpolation algorithm applied to the ion chamber detector array. The information may also provide guidance when choosing a method for interpolating new radiotherapy dosimetry data points.

## Materials and methods

### Octavius and compass dosimetry devices

**Figure 1:**
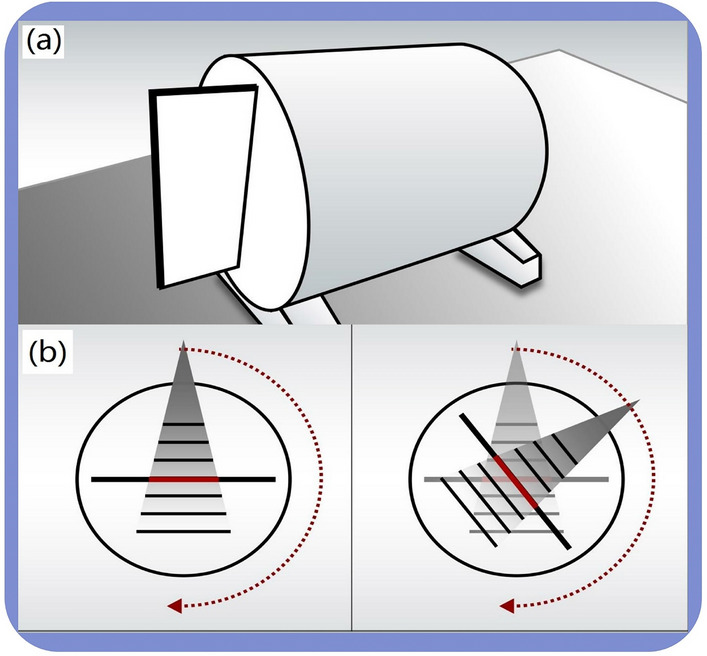
Schematic diagram of Octavius devices. (**a**) Octavius ion chamber detector array was inserted into the cylindrical phantom placed on the treatment couch; (**b**) The detector array always perpendicular to the beam axis measures plane dose at each control point.

**Figure 2:**
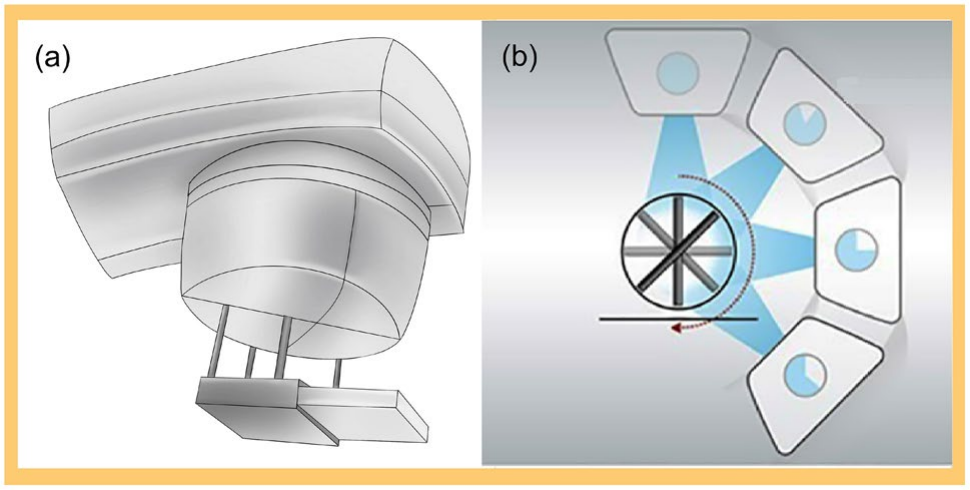
Schematic diagram of Compass devices. (**a**) The MatriXX detector array is mounted on the linac head inside a frame. (**b**) Treatment planning is delivered with the centre of MatriXX at the isocenter in the holder, keeping the array normal to the beam axis during delivery.

In the study, there are two dosimetry divices were used. First, both Octavius 729 and 1500 ion chamber detector arrays (PTW, Freiburg, Germany) were placed into the central cavity of a water-equivalent cylindrical phantom (Fig. 1a)^10^. To synchronize the phantom with the linac, an inclinometer was attached to the linac gantry that provided constant feedback on the gantry angle and kept the detector array always perpendicular to the beam axis (Fig. 1b)^10^. Second, Compass (v.3.1, IBA, Schwarzenbruck, Germany) dosimetry system enables dose reconstruction on patient anatomy using measured dose by a MatriXX detector array^9,20^, which is mounted in a holder attached to the linac gantry with a source-to-detector distance of 76.2 cm to ensure rigid rotation with the gantry (Fig. 2a). When delivering treatment plans, the detector was kept normal to the beam axis in the holder with the center of MatriXX at the isocenter (Fig. 2b). Solid water slabs of 5 cm thickness were placed on top of the detector to add extra build-up and remove electron contamination. Table 1 lists the specifications.

**Table 1:** Specifications of Octavius 729, Octavius 1500, and MatriXX ion chamber detector arrays.

Characteristics	Octavius 729	Octavius 1500	MatriXX
Detector type	Vented parallel plate ion chamber	Vented parallel plate ion chamber	Vented parallel plate ion chamber
Detector number	729	1405	1020
Detector diameter	5 mm	4.4 mm	4.4 mm
Detector shape	Square	Rectangle	cylindrical
Detector height	5 mm	3 mm	5.5 mm
Detector volume	0.125 cm^3^	0.058 cm^3^	0.07 cm^3^
Centre spacing	10 mm	7.07 mm	7.62 mm
Maximum dose rate	12 Gy/min	12 Gy/min	12 Gy/min
Working voltage	400 V	400 V	500 V
Maximum measuring area	27 × 27 cm^2^	27 × 27 cm^2^	24 × 24 cm^2^
Detector shape	Cuboid	Cuboid	Cylindrical
Absorber material on top of the array	5 mm polystyrene	5 mm polystyrene	3 mm ABS resin

### Case selection, treatment planning system, and linacs

In the study, there are two TPS and linacs are used, respectively. First, 40 RapidArc treatment plans involving multiple anatomical sites (17 head/neck, 14 mediastinum/lungs, 9 abdomen/pelvis) were retrospectively included for dosimetric evaluation using Octavius arrays. For these plans, the Varian EDGE linac delivered double arcs or partial arcs both clockwise and counter-clockwise. Beam parameter optimization was performed by the photon optimizer (PO, v13.6.23), and the anisotropic analytical algorithm (AAA, v13.6.23) was used by the Eclipse treatment planning system (Varain Medical Systems, Palo Alto, CA, USA) to calculate the final dose with a 2.5 mm grid size. Second, an Elekta Synergy linac (Elekta Oncology Systems, Stockholm, Sweden) delivered 43 VMAT plans with double/multiple partial arcs involving diverse anatomical locations (11 head/neck, 12 mediastinum/lungs, 20 abdomen/pelvis), which were measured using a MatriXX array. The Oncentra MasterPlan (v4.0, Nucletron B.V., Veenendaal, NL) treatment planning system was used for all delineation and dose calculations.

### Ethical approval and informed consent

All experimental protocols were approved by the Ethics Committee of the Gansu Provincial Hospital (NO. ChiCTR2100054530). The requirement for patient informed consent was waived by the Ethics Committee of the Gansu Provincial Hospital. The methods were carried out according to the relevant guidelines and regulations. Study procedures complied with the Helsinki Declaration of 1964 and the ethical standards of the institutional and/or national research committees, and its later amendments or comparable ethical standards.

## Dose reconstruction

### Octavius reconstruction

**Figure 3:**
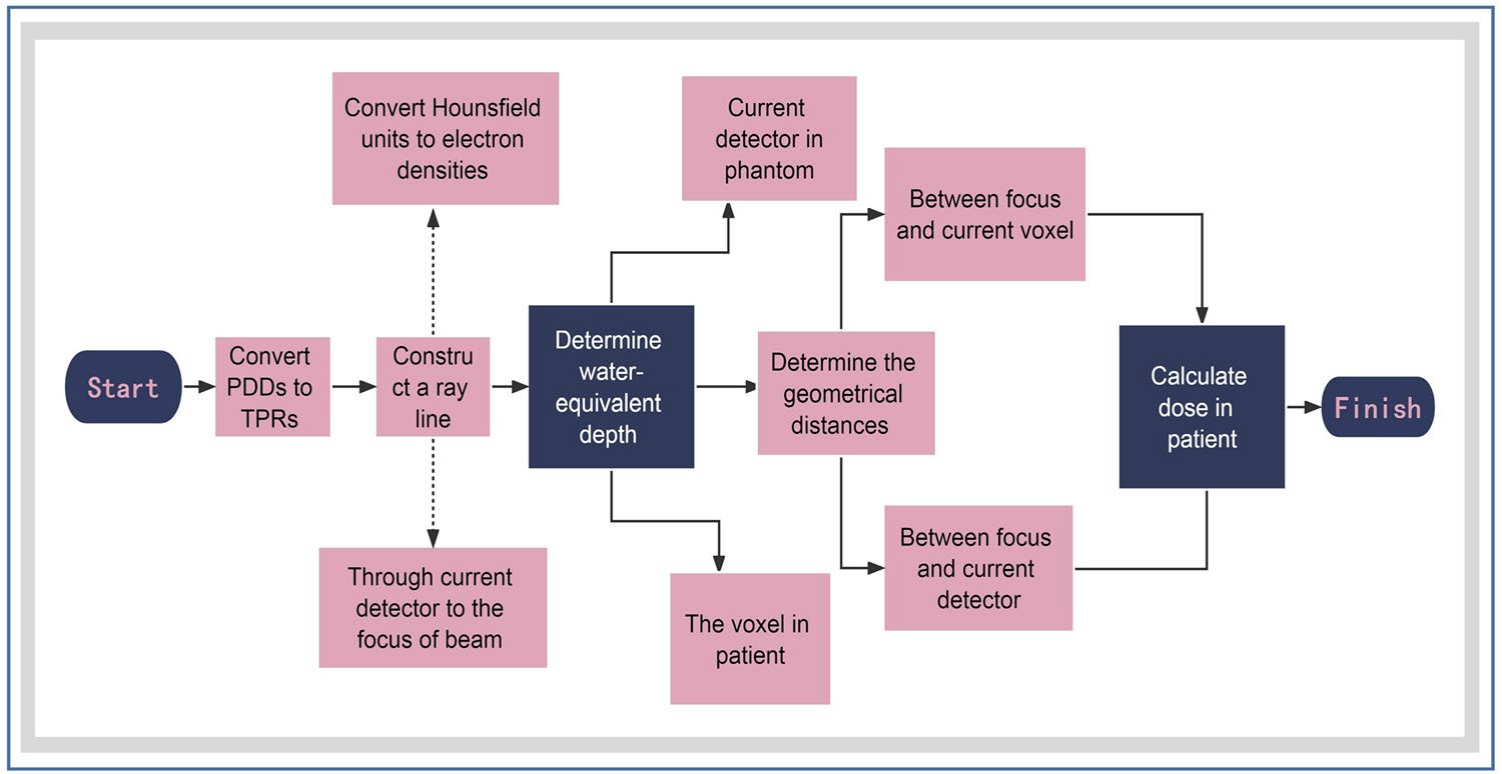
Flow chart of dose reconstruction on CT images by Octavius arrays. The 3D dose distribution within the phantom was calculated using percent depth dose data at different beam fields (fields of 4 × 4 cm^2^, 10 × 10 cm^2^, and 26 × 26 cm^2^ are mandatory, others may slightly contribute to the accuracy) by linear interpolation. Based on this, the DVH on the patients’ anatomical structures was calculated. PDDs = percent depth doses; TPRs = tissue phantom ratios.

By linear interpolation, the phantom dose can be reconstructed from the plane measurement dose, followed by the RT plan, RT structures, RT dose, and CT datasets being imported into the Verisoft software (v7.1). To determine the dose on a ray passing through a point in the anatomical site, the relationship between the dose measured by the current detector in the phantom and the dose at the corresponding point on the ray in the CT image was established^10^. Subsequently, DVHs were calculated based on the reconstructed dose in the regions of interest, and the flow chart was shown in Fig. 3.

### Compass reconstruction

Compass uses a dedicated beam model to create a virtual accelerator and describe the linac characteristics (e.g., energy spectrum, lateral beam quality variations, and collimators)^21^. Plane measured dose were acquired by Compass software with a resolution of 1 cm at the isocenter^21^. Combined with the modelʼs predicted response^20,21^, linear interpolation was used to reconstruct the plane dose at a spatial resolution of 2 mm the same as the TPS grid resolution. After importing all digital imaging and communications in medicine files from 43 treatment plans into Compass, the 3D dose distribution on CT images was reconstructed based on a collapsed cone convolution/superposition algorithm (CCC)^20,21^.

**Figure 4:**
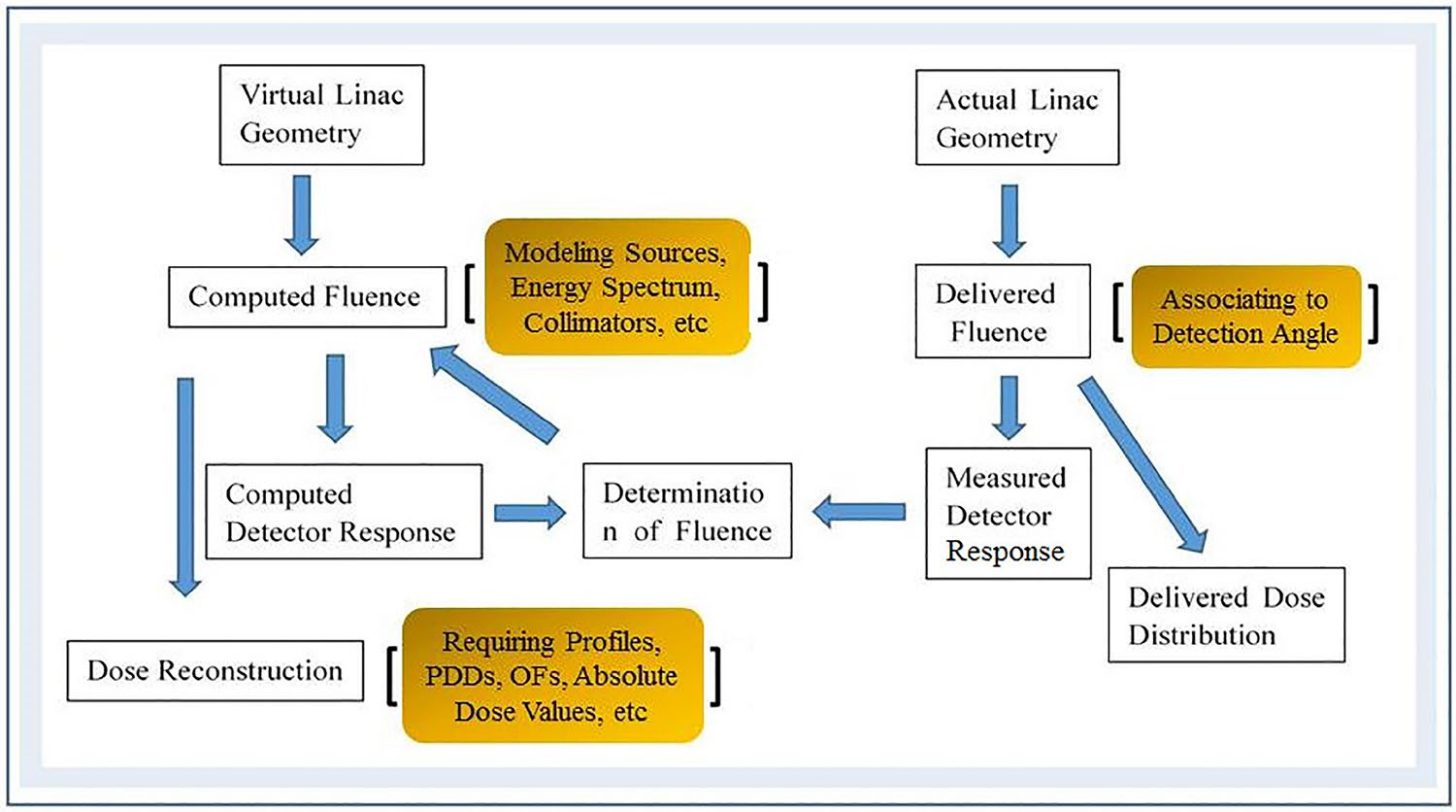
A diagram illustrating the dose reconstruction using the Compass dosimetry system. Compass determines the fluence correction by comparing computed and measured detector responses. Thus, the reconstructed dose includes a fluence correction factor. OFs are output factors.

Compass software can predict the detector response by a detector model and a response calculation algorithm^21^. The differences between the predicted and measured detector responses were used as an input to the final dose calculation^21^. Figure 4 describes the reconstruction method.

**Figure 5:**
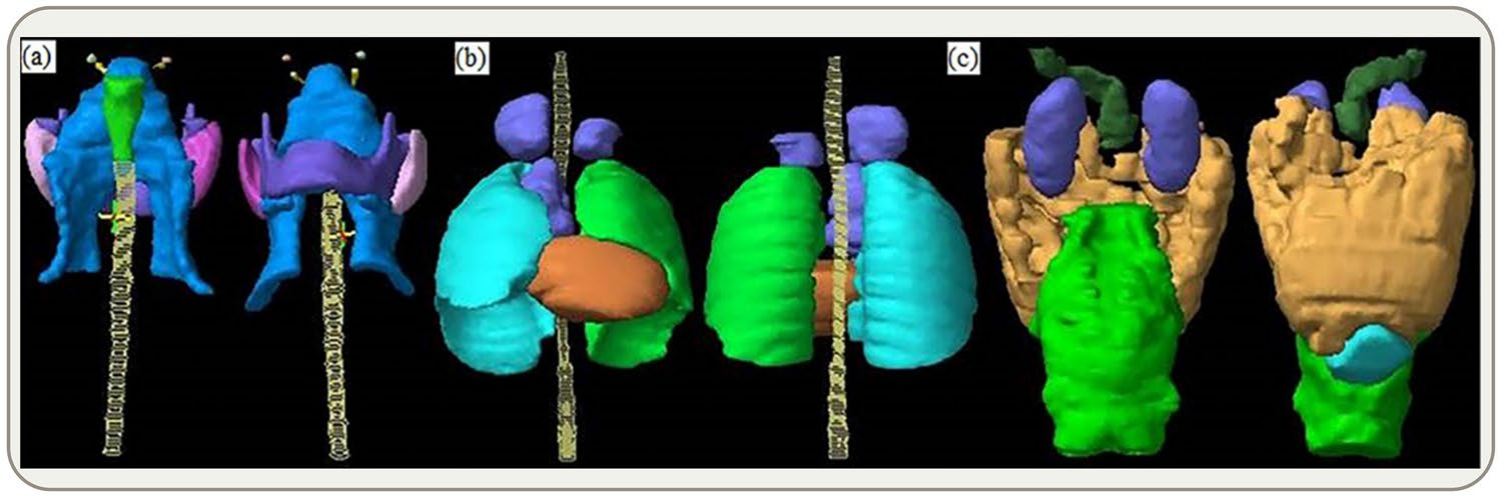
Radiation oncologists delineate PTVs and OARs on CT images. (**a**) PTVs are in blue, while the remaining colors indicate OARs; (**b**) PTVs are in light coffee, while the remaining colors indicate OARs; (c) PTVs are in light brown, while the remaining colors indicate OARs.

Figure 5 illustrates PTVs and OARs at different anatomical locations that were contoured according to the contouring guidelines of the Radiation Therapy Oncology Group (RTOG) as described in the referred literatures^22–25^. To be clinically acceptable, a plan must cover 100% of the prescribed dose for at least 95% of the PTVs^26^. The calculated and reconstructed doses were compared by determining the differences in DVH metrics. Recommendations of the International Commission on Radiation Units and Measurements (ICRU) report 83 were used to extract evaluation parameters for PTVs (*D*_98_, *D*_2_, *D*_50_, and *D*_mean_) and maximum/mean doses (*D*_max_/*D*_mean_) for OARs, in which *D*_2_ is recommended to represent the approximate maximum dose for tandem OARs^27^. *D*_98_, *D*_2_, and *D*_50_ are the doses covering 98%, 2%, and 50% of the PTVs/OARs volume^28,29^, respectively. ${D}_{\mathrm{reconstruction}}$ and *D*_TPS_ represent reconstructed and calculated doses, respectively. The absolute value of the percentage dose deviation *D*_D_ (%) is defined as

1 $${D}_{\mathrm{D}}\left(\mathrm{\%}\right)=\left|\left({D}_{\mathrm{reconstruction}}-{D }_{\mathrm{TPS}}\right)/{D}_{\mathrm{TPS}}\right|\times 100\%$$

Dosimetric metrics of partial organs at risk were evaluated by the percentage volume of the considered OARs receiving a specific dose or less (in %)^28^, and the absolute value of percentage volume deviation *V*_D_ (%) is defined as

2 $${V}_{\mathrm{D}}\left(\mathrm{\%}\right)=\left|\left({V}_{\mathrm{reconstruction}}-{V}_{ \mathrm{TPS}}\right)/{V}_{ \mathrm{TPS}}\right|\times 100\%$$

${V}_{\mathrm{reconstruction}}$ is the reconstructed volume receiving a dose, and ${V}_{ TPS}$ is the TPS volume receiving the same dose.

## Interpolation and analysis

### Interpolation types

A volumetric dose can be created by interpolating all delivered planar doses. Potential interpolation methods are usually based on geometric shapes, including squares, triangles, rectangles or cylinders^19^. Herein, phantom shape for Octavius and Compass determined the selection of linear interpolation over the cylindrical and rectangle geometry methods (both unilinear and bilinear interpolations were included), respectively. Each of the measurements produced a maximum delivered dose matrix with dimensions of 27 × 27 cm^2^ and 24 × 24 cm^2^, respectively. To create the interpolated volumetric dose, all the delivered planar doses were evaluated and interpolated. During arc beam delivery, 729 and 1500 detector arrays were inserted into the phantom to measure doses on each control point with gantry rotation (a full arc beam rotated 360 degrees, containing 178 control points), to produce ≤ 729 and ≤ 1405 discrete data points, respectively. For the MatriXX array, a full arc beam was set to 120 control points, each generating ≤ 1020 discrete data points. Measured doses at all control points can be interpolated to create a volume dose with a certain voxel size. Every two adjacent ion chambers along a row of detectors refer to unilinear interpolation, and the re-interpolation of interpolated data points on all control points along the linac rotation directions can indicate bilinear interpolation. Measurements can be performed along 360 degrees in both directions (*x* and *y*), which involves a transformation between the cartesian (*x, y, z*) and cylindrical (*r,*$\varphi ,\; z$) coordinates that is given by the following equations:

3 $$ x = r\cos \varphi ,\;y = r\sin \varphi ,\; z = z,\;r = \sqrt {x^{2} + y^{2} } $$

4 $$ \varphi = \left\{ {\begin{array}{*{20}l} 0 \hfill & {if x = 0 and y = 0} \hfill \\ {arcsin\left( \frac{y}{r} \right)} \hfill & { if x \ge 0} \hfill \\ { - arcsin\left( \frac{y}{r} \right) + \pi } \hfill & {if x < 0} \hfill \\ \end{array} } \right. $$

**Figure 6:**
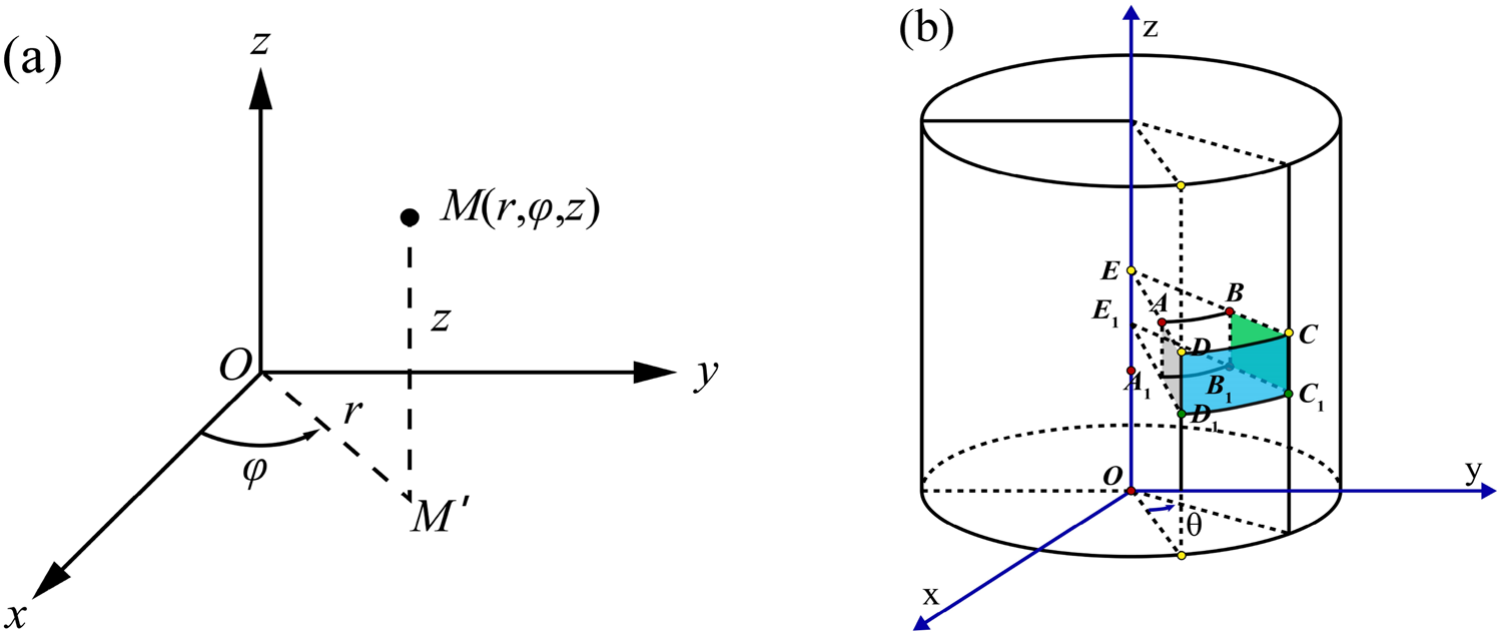
(**a**) Coordinate transformation between cylindrical and cartesian variables; (**b**) Schematic presentation of data point interpolation during gantry rotation along with ion chamber detector arrays.

In Fig. 6a, the three variables in the cylindrical coordinates are *r*, $\varphi , \mathrm{and} z$, where cylindrical coordinates have a variable $z$ as well as cartesian coordinates and r is the distance between origin point o and projection point *M'* of point *M* in plane xoy. For 729 and 1500 arrays, $r\in \left[\mathrm{0,13.5}\right]$. *φ* is the angle rotated from the x-axis to OM' counter-clockwise, $\varphi$∈$\left[0,2\uppi \right]$, *z* $\in \left[\mathrm{0,27}\right]$. $r\in \left[\mathrm{0,12}\right]$ and *z* $\in \left[\mathrm{0,24}\right]$ for the MatriXX array. In Fig. 6b, the array is shown rotating at an angle ɵ by the x-axis (corresponding to gantry moving from one control point to another), and points A to B, A_1_ to B_1_, D to C, and D_1_ to C_1_ imply cylindrical geometry linear interpolation, namely, interpolation in cylindrical coordinates. Points E to E_1_, A to A_1_, B to B_1_, C to C_1_, D to D_1_, A to E, D to A, A_1_ to E_1_, D_1_ to A_1_, B to E, B_1_ to E_1_, and C_1_ to B_1_ are two-point linear interpolations.

### Linear interpolation

Linear interpolation along a row of ion chambers can be constructed as follows: given that the $y=f(x)$ function has function values ${y}_{0}{=f({x}_{0}), y}_{1}=f\left({x}_{1}\right)$ at nodes ${x}_{0}$ and ${x}_{1}$, the polynomial $\varphi (x)={a}_{0}+{a}_{1}x$ is constructed so that $\varphi \left({x}_{0}\right)={y}_{0}, \varphi \left({x}_{1}\right)={y}_{1}$. This is linear interpolation, and the interpolation error on nodes is 0. The linear interpolation formula between two discrete dose points, namely, the line through $\left( {x_{0} ,\;y_{0} } \right)$ and $\left( {x_{1} ,\;y_{1} } \right),\;$ can be expressed as^13^

5 $$\varphi \left(x\right)=\frac{x-{x}_{1}}{{x}_{0}-{x}_{1}}{y}_{0}+\frac{x-{x}_{0}}{{x}_{1}-{x}_{0}}{y}_{1}$$

Linear interpolation only uses two points, which is easy to calculate. The smaller the interpolation interval $\left[{x}_{0}, {x}_{1}\right]$ is, the smaller the error between $f(x)$ and $\varphi \left(x\right)$^13^is. Accordingly, the more uniform the calculated dose of PTVs and OARs, the more accurate the reconstructed dose should be, while the dose drop regions outside the field will introduce a larger interpolation error.

### Bilinear interpolation

**Figure 7:**
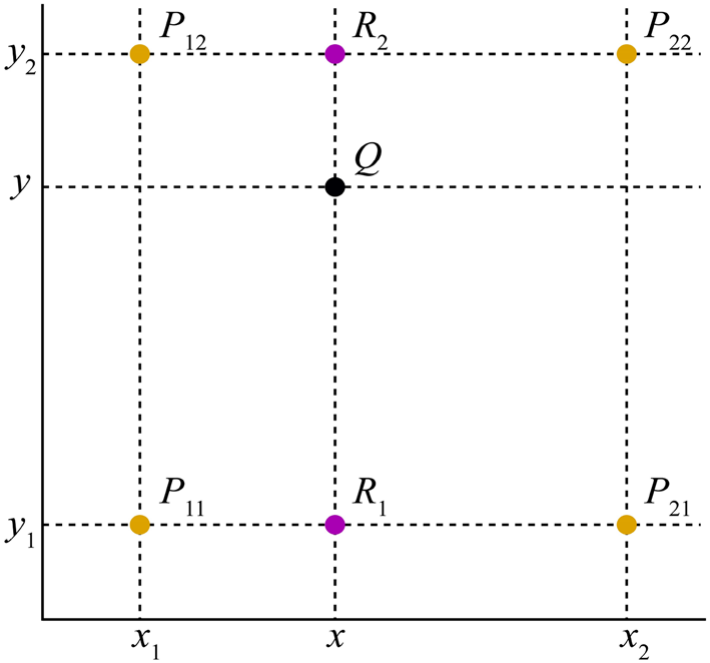
Schematic graph of bilinear interpolation.

Due to the arrangement of individual ion chambers and centre spacing, bilinear interpolation along the detector plane may be required to ensure that the spatial resolutions of the evaluated dose distribution are not lower than the TPS dose distribution. Bilinear interpolation is a linear extension of bivariate interpolation, performed in two directions^30^. Every interpolation is linear in sampling values and positions, however, the total interpolation is nonlinear, as shown in Fig. 7. Yellow points represent the measurement points at single ion chambers, whereas a black point represents the point to be interpolated. Ion chamber dose for function *f* at four points *P*_11_ = (*x*_1_, *y*_1_), *P*_12_ = (*x*_1_, *y*_2_), *P*_21_ = (*x*_2_, *y*_1_), and *P*_22_ = (*x*_2_, *y*_2_) are known. To obtain the value of function *f* at point *Q* = (*x*, *y*), the first step is to obtain R_1_ and R_2_ by linear interpolation in the x direction. Next, interpolation in the y direction is performed to obtain the point Q dose, which is the point between adjacent ion chamber gaps, and then, *f* (*x*, *y*) is obtained.

Linear interpolation in the x direction: *R*_1_ was obtained by interpolation on the measured doses of ion chambers at points *P*_11_ and *P*_21_, while *R*_2_ was obtained by interpolations of ${P}_{12}$ and ${P}_{22}$. This is summarized in Eqs. (6) and (7)^30^

6 $$f\left({R}_{1}\right)\approx \frac{{x}_{2}-x}{{x}_{2}-{x}_{1}}f({P}_{11})+\frac{x-{x}_{1}}{{x}_{2}-{x}_{1}}f({P}_{21})\mathrm{ where }{R}_{1}=\left(x,{y}_{1}\right)$$

7 $$f\left({R}_{2}\right)\approx \frac{{x}_{2}-x}{{x}_{2}-{x}_{1}}f({P}_{12})+\frac{x-{x}_{1}}{{x}_{2}-{x}_{1}}f({P}_{22})\mathrm{ where }{R}_{2}=\left(x,{y}_{2}\right)$$

The dose at point Q is calculated by interpolating points *R*_1_ and *R*_2_ in the Y direction.

8 $$f\left(\mathrm{Q}\right)\approx \frac{{y}_{2}-y}{{y}_{2}-{y}_{1}}f({R}_{1})+\frac{y-{y}_{1}}{{y}_{2}-{y}_{1}}f({R}_{2})$$

In fact, the value at Q point is equal to the weighted average area of the four diagonal rectangles formed by the Q point and the surrounding points. A linear interpolation method is used to interpolate one-dimensional data, which uses the left and right adjacent points to be interpolated. A weighted average algorithm can be used to calculate the *y* value at Q point by combining points R_1_ and R_2_. The *y* value is calculated mainly according to the distance between the calculated point and the two endpoints in the *y* direction. Specifically, an interpolated point is assigned specific gravity based on its distance from two endpoints. To interpolate measured data points into 0.25 × 0.25 × 0.25 cm^3^ resolution, the distances between interpolated points and left/right adjacent data points were equal for the 729 array, thus, the same specific gravity is assigned. For the 1500 array, however, the 0.707 cm centre distance determines the unequal distance between the interpolated and the left/right points, thus, the proportion is different. To interpolate data points into 0.20 × 0.20 × 0.20 cm^3^ resolution by the MatriXX array, the interpolated points and left/right data points are unequally spaced due to the 1 cm distance between the adjacent measured points at the isocentre position. Consequently, the proportion may be different as well.

### Fundamental assumptions

The linear interpolation method for creating volumetric doses may cause errors associated with the interpolation algorithm itself that are not present in treatment planning. Herein, interpolated dose points are created from multiple plane measurements in cylindrical and rectangle patterns. Thus, spatial sampling should play a crucial role in the interpolation accuracy. It is possible to achieve better interpolation results using a detector array with a smaller centre spacing.

### Statistics inference testing

For Octavius dosimetry, absolute percentage dose/volume deviations (Pdd/%) of PTVs and OARs were plotted using Grad Prism 9.1 software. The Shapiro–Wilk significance hypothesis test was performed on each data group of 729 and 1500 arrays. To determine whether differences in Pdd/% averages in both normally distributed data groups were significant, a paired *t*-test was used at a 5% significance level. Otherwise, the Wilcoxon signed rank test was used. A *P* value of less than 0.05 was considered statistically significant. Compass results were described as the mean ± standard deviation (SD).

## Results

### Absolute percentage dose/volume deviations obtained from Octavius 729 and 1500 arrays

**Figure 8:**
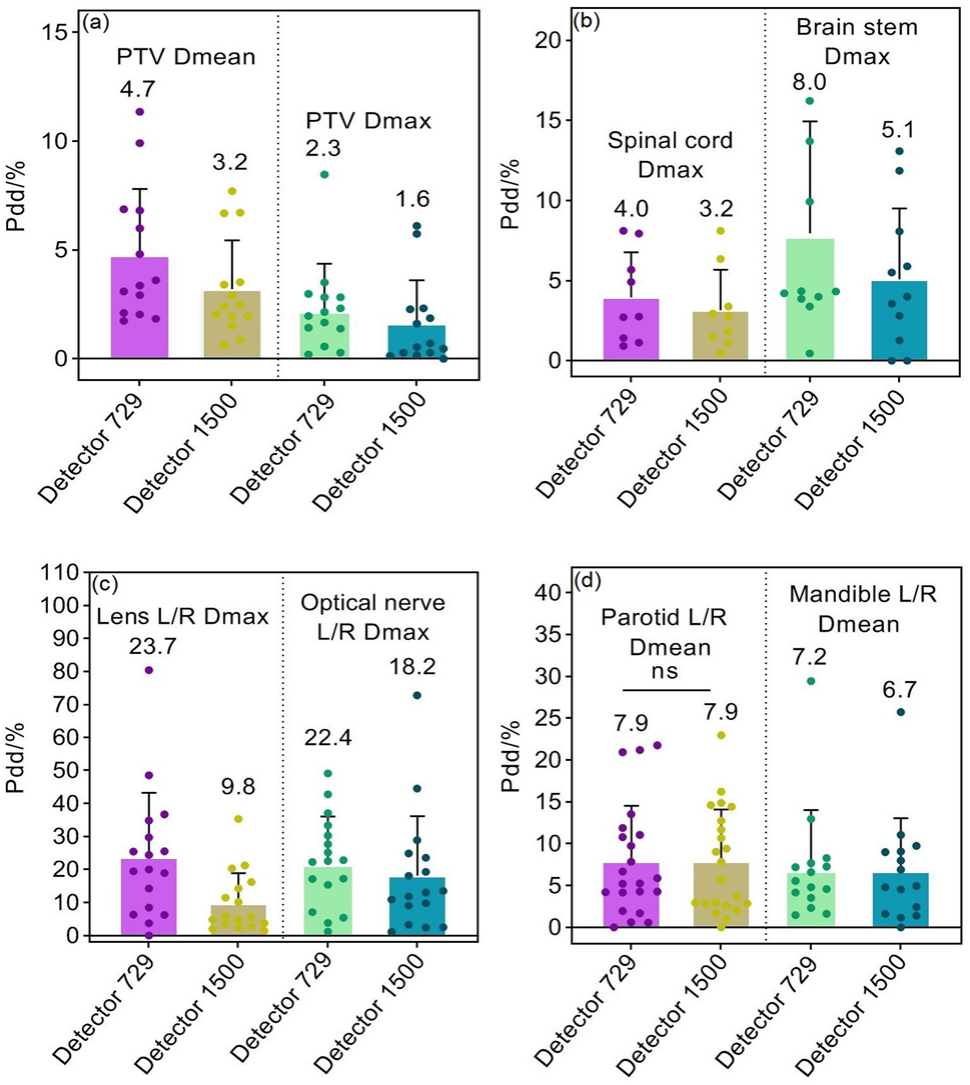
Pdd/% for head and neck radiotherapy plans. (**a**) Percent dose deviations of mean and maximum dose in PTVs; (**b**) Percent dose deviations of maximum dose in spinal cord and brainstem; (**c**) Percent dose deviations of maximum dose in bilateral lens and optic nerves; (**d**) Percent dose deviations of mean dose in bilateral parotid gland and mandible. Pdd/% = Percent dose/volume deviation; ns: *p* > 0.05.

**Figure 9:**
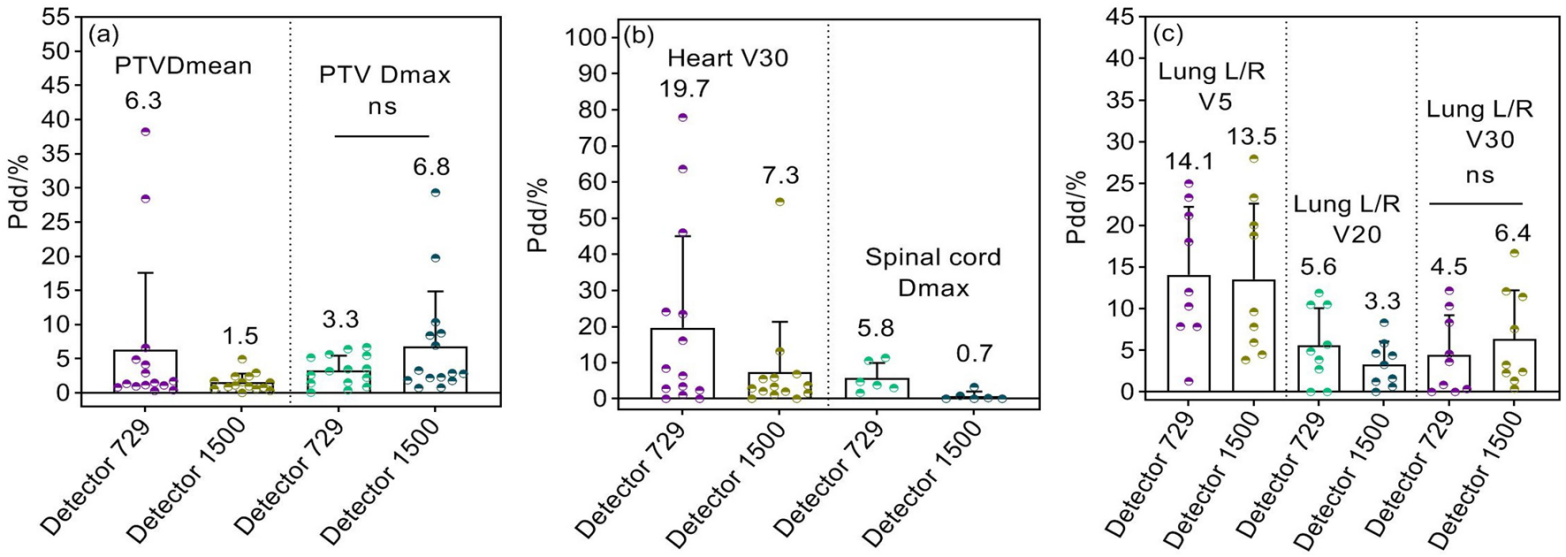
Pdd/% for mediastinum and lung plans. (**a**) Percent dose deviations of mean and maximum doses in PTVs; (**b**) Percent volume deviations of heart exposed to a dose of 30 Gy, and percent dose deviation of maximum dose in spinal cord; (**c**) Percent volume deviations of bilateral lungs exposed to a dose of 5 Gy, 20 Gy, and 30 Gy, respectively.

**Figure 10:**
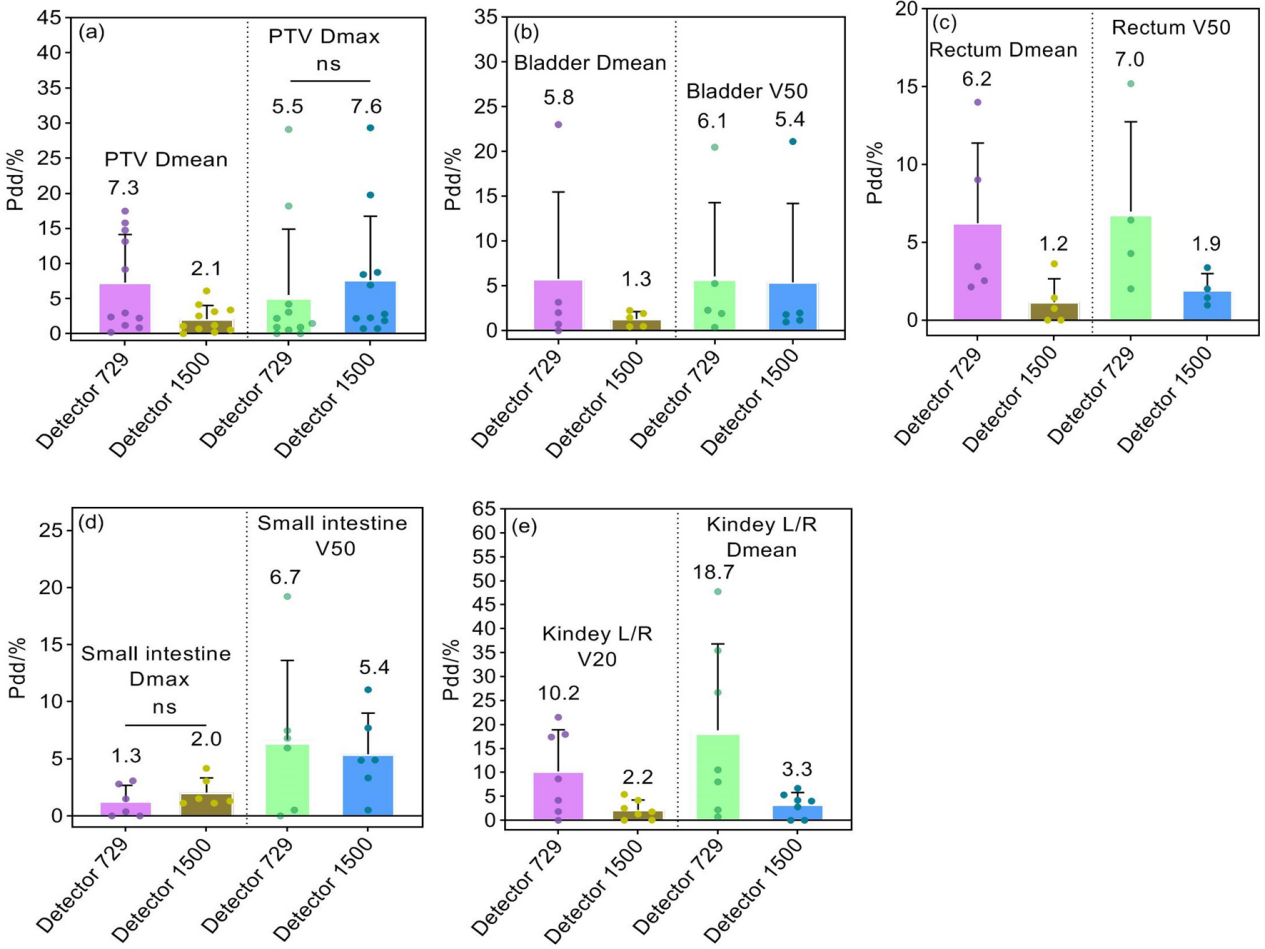
Pdd/% for abdomen and pelvis plans. (**a**) Percent dose deviations of mean and maximum dose in PTVs; (**b**) Percent dose deviations of mean dose and percent volume deviations exposed to a dose of 50 Gy in bladder; (**c**) Percent dose deviations of mean dose and percentage volume deviations exposed to a dose of 50 Gy in rectum; (**d**) Percent dose deviations of maximum dose and percentage volume deviations exposed to a dose of 50 Gy in small intestine; (**e**) Percent dose deviations of mean dose in bilateral kidneys and percentage volume deviations of bilateral kidneys exposed to a dose of 20 Gy.

DVH calculation is only valid for anatomical regions within the measurement range of arrays (27 × 27 cm^2^). By comparing calculated and reconstructed DVHs, absolute percentage dose/volume deviations (Pdd/%) and the averages of partial dosimetric indices for PTVs and OARs are shown in Figs. 8, 9 and 10. The Pdd/% averages of D_mean_ for PTVs reconstructed by 729 and 1500 arrays range between 4.7 and 7.3% and between 1.5 and 2.3%, while the Pdd/% averages of D_max_ demonstrate ranges of 2.3–5.5% and 1.6–7.6%, respectively. The Pdd/% averages of OARs vary from 1.3% (95% confidence interval (CI): 0.7–1.6%) to 23.7% (95% CI:18.9–26.3%) and from 0.7% (95% CI: 0.4–1.1%) to 18.2% (95% CI:15.7–20.9%) for 729 and 1500 arrays, respectively. There were several outliers: Pdd/% averages of D_mean_ in parotid glands were equal (Fig. 8d); Pdd/% averages in partial PTVs and OARs reconstructed by the 1500 array were slightly higher, such as D_max_ of PTVs (Figs. 9a and 10a), percent volume deviations of left/right lungs exposed to a dose of 30 Gy (Fig. 9c), and D_max_ of small intestines (Fig. 10d); however, differences were insignificant (*p* > 0.05). As a result, the reconstructed dose by the 1500 detector array is closer to the TPS dose relative to that of the 729 detector. As expected, a smaller detector spacing leads to a more accurate 3D dose reconstruction. Of note, there is a lower spread out of Pdd/% values in discrete distributions for the 1500 array than for the 729 array in most datasets. This may be explained by the fact that the detector resolution is higher, more data points are measured, and fewer points are estimated by interpolation, consequently, the introduced dose estimation error is less. Compared to other OARs, the Pdd/% averages in D_max_ in the lens and optic nerve were larger (> 20%). The maximum Pdd% value in the 729 array was 80.39% (lens), while in the 1500 array, 72.81% (left/right optical nerve) was the maximum. Specifically, the Pdd/% average in D_max_ in the lens reconstructed by the 1500 array is 8.7%, while that reconstructed by the 729 array is 23.7%. At rest, the lens is 9 to 10 mm in diameter, 4 to 5 mm in thickness, and approximately 0.2 cm^3^ in volume, while the optic nerve is 4 to 6 mm in diameter and 4 to 9 mm in length. For the 5 mm edge gap of adjacent detectors in the 729 array, the small volumes of the optic nerve and lens increase the probability that it is in the gap of adjacent ion chambers and cannot be sampled or only a small partial can be sampled, while the smaller gap in the 1500 array may contribute to relatively more sampling. In addition, small volumes are easily affected by factors such as phantom positioning, and fewer measured points may result in more errors in dose calculation.

### Absolute percentage dose/volume deviations obtained from the compass dosimetric system

The relative differences in DVH metrics for the planning target volumes and OARs are listed in Table 2. For D_95%_ and D_mean_ of the planning target volumes, the maximum Pdd/% average was 3.7%, the minimum SD was less than 0.5%, and the maximum SD was not more than 2.6%. For OARs, the minimum Pdd/% average was less than 0.3%, and the maximum Pdd/% average was close to 6.7%. Among them, the lens and optic nerve cause the largest SD value (near 9.0%), and the maximum Pdd/% averages are 6.67% and 6.03% for V_5_ and D_mean_ of lung L/R, respectively. The results on the regions of interest of gastric and pelvic tumors showed that the maximum Pdd/% average was 5.04% for the bilateral kidney, while the Pdd/% averages of other regions of interest were almost less than 3%, and the maximum standard deviation did not exceed 3.5%.

**Table 2:** Dosimetric differences in 43 VMAT plans at various anatomical sites (%, means ± SD).

Anatomical structures	DVH metrics	Pdd (%)	CI (95%)	Anatomical structures	DVH metrics	Pdd (%)	CI (95%)
**Head neck** CTV1-P	D_95%_ (Gy)	1.20 ± 1.50	[0.97, 1.46]	**Gastric** PGTV	D_95%_ (Gy)	2.32 ± 1.27	[1.97, 2.62]
	D_mean_ (Gy)	0.40 ± 1.67	[0.34, 0.96]		D_mean_ (Gy)	1.46 ± 1.13	[1.21, 1.99]
CTV2-P	D_95%_ (Gy)	1.35 ± 1.60	[1.07, 1.66]	PTV	D_95%_ (Gy)	0.87 ± 1.24	[0.67, 1.36]
	D_mean_ (Gy)	0.65 ± 1.70	[0.39, 1.06]		D_mean_ (Gy)	1.01 ± 0.93	[0.86, 1.36]
CTV-NR-P	D_95%_ (Gy)	1.32 ± 1.06	[1.09, 1.44]	Liver	V_30_ (%)	0.83 ± 1.63	[0.59, 1.12]
	D_mean_ (Gy)	0.32 ± 1.46	[0.21, 0.78]		D_mean_ (Gy)	1.29 ± 2.55	[1.03, 1.98]
CTV-NL-P	D_95%_ (Gy)	1.35 ± 2.60	[1.01, 1.72]	Stomach	V_30_ (%)	2.59 ± 1.95	[1.97, 2.76]
	D_mean_ (Gy)	0.85 ± 1.81	[0.55, 1.31]		D_max_ (Gy)	2.88 ± 1.58	[2.49, 3.17]
Brain stem	D_1%_ (Gy)	3.07 ± 3.27	[2.67, 3.85]	Pancreas	V_30_ (%)	1.68 ± 1.62	[1.22, 1.96]
	D_max_ (Gy)	3.93 ± 2.65	[3.12, 4.15]		D_mean_ (Gy)	2.46 ± 2.43	[2.01, 2.98]
Parotid L/R	D_mean_ (Gy)	2.97 ± 5.08	[3.16, 4.07]	Spleen	V_30_ (%)	1.18 ± 1.45	[0.89, 1.55]
	V_30_ (%)	4.90 ± 1.62	[4.31, 5.21]		D_mean_ (Gy)	2.03 ± 1.17	[1.72, 2.35]
Len L/R	D_max_ (Gy)	4.59 ± 8.89	[3.93, 5.89]	Kidney L/R	V_20_ (%)	3.19 ± 2.95	[2.66, 3.63]
Optical nerve L/R	D_max_ (Gy)	4.98 ± 8.03	[4.02, 5.20]		D_mean_ (Gy)	5.04 ± 3.45	[4.17, 5.63]
Mandible L/R	D_mean_ (Gy)	2.97 ± 5.09	[2.23, 3.77]	Spinal Cord	D_1%_ (Gy)	2.09 ± 1.26	[1.68, 2.28]
	V_30_ (%)	0.21 ± 6.67	[0.13, 1.82]		D_max_ (Gy)	2.50 ± 1.55	[2.02, 2.98]
Spinal Cord	D_1%_ (Gy)	1.80 ± 2.74	[1.23, 2.02]	**Prostate** PTV Pr	D_95%_ (Gy)	2.43 ± 0.38	[2.04, 2.51]
	D_max_ (Gy)	2.09 ± 2.63	[1.56, 2.46]		D_mean_ (Gy)	1.66 ± 0.68	[1.43, 1.74]
**Chest** PGTVnd	D_95%_ (Gy)	1.20 ± 1.50	[1.01, 1.76]	PTV SV	D_95%_ (Gy)	0.96 ± 1.03	[0.77, 1.15]
	D_mean_ (Gy)	0.40 ± 0.80	[0.28, 0.45]		D_mean_ (Gy)	1.01 ± 0.85	[0.92, 1.31]
PTV	D_95%_ (Gy)	3.70 ± 1.60	[3.03, 4.11]	Rectum	V_50_ (%)	1.64 ± 0.63	[1.45, 1.77]
	D_mean_ (Gy)	1.00 ± 1.00	[0.88, 1.52]		D_mean_ (Gy)	2.72 ± 0.48	[2.35, 3.01]
Lung L/R	V_5_ (%)	6.67 ± 0.66	[6.23, 7.06]	Bladder	V_50_ (%)	2.67 ± 1.48	[2.11, 2.88]
	V_20_ (%)	1.78 ± 1.54	[1.09, 2.01]		D_mean_ (Gy)	1.07 ± 0.48	[1.01, 1.26]
	V_30_ (%)	1.80 ± 1.38	[1.45, 2.03]	Colon	V_50_ (%)	0.61 ± 0.58	[0.51, 0.71]
	D_mean_ (Gy)	6.13 ± 1.13	[5.77, 6.53]		D_max_ (Gy)	1.04 ± 0.05	[0.98, 1.06]
Heart	V_30_ (%)	4.09 ± 1.14	[3.51, 4.35]	Femoral L/R	V_50_ (%)	0.91 ± 0.55	[0.77, 1.25]
	D_mean_ (Gy)	3.83 ± 1.13	[3.24, 4.01]		D_mean_ (Gy)	2.41 ± 0.43	[2.29, 2.69]
Esophagus	V_40_ (%)	0.44 ± 1.35	[0.29, 0.62]	Small intestine	V_50_ (%)	1.11 ± 0.75	[0.93, 1.56]
	D_mean_ (Gy)	3.39 ± 1.33	[2.99, 3.79]		D_max_ (Gy)	1.69 ± 0.95	[1.38, 2.01]
LAD	D_mean_ (Gy)	2.33 ± 3.53	[1.78, 3.01]	Pelvic bone	V_20_ (%)	3.77 ± 1.38	[3.32, 4.11]
Trachea	V_40_ (%)	1.79 ± 1.69	[1.41, 2.21]		V_30_ (%)	0.29 ± 0.65	[0.22, 0.56]
	D_mean_ (Gy)	3.09 ± 3.12	[2.48, 3.89]		D_mean_ (Gy)	2.72 ± 1.39	[2.27, 3.16]

CTV1-P is the planning target volume of the high-risk clinical tumor area of the primary tumor; CTV2-P is the planning target volume of the low-risk clinical tumor area of the primary tumor; CTV-NR-P is the planning target volume of the clinical tumor area of the right lymph node; CTV-NL-P is the planning target volume of the clinical tumor area of the left lymph node. D_95%_, D_1%_ = minimum dose received by 95% and 1% of the planning target volume, respectively; D_mean_ = mean dose; V_5_, V_20_, V_30_, V_40_, V_50_ = volume of the considered organ receiving 5 Gy, 20 Gy, 30 Gy, 40 Gy, and 50 Gy or less (in %). Pr = prostate; SV = seminal vesicles; PLN = pelvic lymph nodes; PTV is planning target volume; PGTV is planning gross tumor volume; PGTVnd is planning gross target volume (positive lymph node); Pdd/% = percent dose deviation; CI indicates confidence interval of Pdd/% averages.

### Small local tissue density inhomogeneity results in superior dose reconstruction in anatomical regions

**Figure 11:**
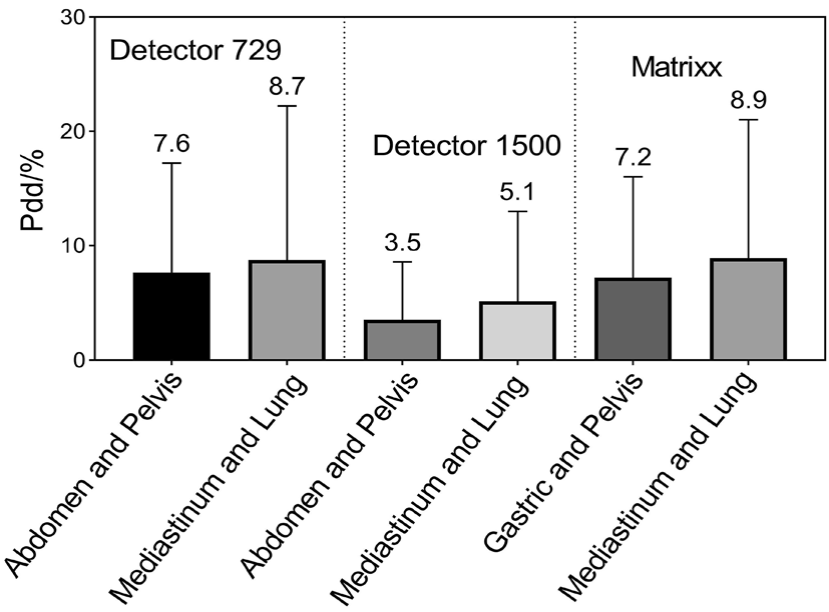
Bar charts display Pdd/% results at various anatomical sites.

For the same detector array, Fig. 11 shows that the Pdd/% averages of mixed PTVs and OARs at abdomen/gastric and pelvic sites are relatively smaller, while plans of mediastinum and lung showed slightly larger Pdd/% averages. Thorax structures contain lung tissue whose mass density is close to air and less than water, compared to pelvic structures, indicating that in regions with high inhomogeneity and discontinuities in local tissue density, such as areas of low density tissue surrounding lung lesions, contribute to inferior accuracy in dose reconstruction.

## Discussions

An acceptable radiotherapy plan can be evaluated by comparing reconstructed and planned DVHs to determine whether the dose differences between them are clinically meaningful. Using Verisoft software, the plane measurement dose is first reconstructed within the phantom by linear interpolation, and on CT images, the voxel dose is calculated from the dose determined in the phantom and electron density at the corresponding depth^31,32^. After the doses for all voxels within the effective measurement range of the detector array were determined, DVHs were calculated using plan parameters at each control point. From the beam modeling process, the Compass dosimetry system reconstructed a plane dose map by fitting electron and photon spectra, beam quality variations, source parameters, tongue and groove, etc. As a consequence, usual TPS commissioning measurements are needed, such as relative distributions (profiles and percent depth dose), output factors, and absolute doses^33^. Herein, DVHs were reconstructed on CT images based on plane doses generated by both dosimetric systems for 83 VMAT treatment plans at different anatomic sites. We compared the reconstruction dose to the TPS dose to investigate: (i) the potential principle of the linear interpolation algorithm in reconstructing the volume dose in the rotational radiotherapy technique; (ii) how different ion chamber spacings and numbers of sampling points affect the accuracy of interpolating new data points; and (iii) the effects of anatomical sites and mass density structures on dose reconstruction.

Based on our results, it appears that the dose reconstructed by the 1500 detector array is more similar to the TPS dose than the 729 detector array. According to previous findings, the 729 detector array resulted in a lower point dose in the drop area than the TPS dose, and the linear interpolation on adjacent data points may introduce sampling uncertainty^15^. Additionally, despite good in-field agreement, the penumbra agreement was poor for open-field measurements and TPS dose analysis in 2D profiles^15^. Possibly, this difference is caused by the linear interpolation between adjacent measurement points, which may lead to underestimation or overestimation of the dose on the interpolated points^15,33^. Combined with our findings, these results showed that detector arrays with lower spatial resolution can introduce more variabilities in interpolating new dose points. As a result, γ pass rate calculations and DVH reconstruction become more uncertain. Linear interpolation looks for patterns in a known data sequence (which can be viewed as a series of discrete points in coordinates) and uses these patterns to numerically estimate regions between adjacent data points, its main application is to reasonably compensate for missing data^19^. The Octavius and Compass dosimetric systems may use both unilinear and bilinear interpolations according to the size and arrangement of single ion chambers when interpolating the measured dose to obtain the volume dose with the same dose grid as the TPS. Other dosimetric software also uses bilinear interpolation^16^. Since two points estimated by unilinear interpolation are interpolated twice to obtain the data points estimated again, compared with unilinear interpolation, bilinear interpolation should cause more errors.

For photon radiotherapy, many dose calculation algorithms are available, with varying degrees of complexity regarding photon scattering, electron transport handling, especially in heterogeneous media, and computation time^34,35^. Specifically, three types of algorithms are defined according to the calculation accuracy: correction-based algorithms (type a), model-based algorithms (type b), and principle-based algorithms (type c)^34,35^. Various commercial TPS use model-based algorithms. In AAA, the 3D pencil beam convolution/superposition algorithm uses a source model consisting of primary bremsstrahlung photons, extra focal photons, and contaminating electrons^3^. AAA uses model parameters with physical significance (such as photon energy spectrum, mean radial energy, and scatter kernels) to describe the characteristics of photon and electron fluence and their energy spectra in the treatment beam^36^. As a result, it is capable of reflecting the actual transport process of ionizing radiation during its calculation. In contrast, Verisoft calculates doses using PDD data specific to field sizes from 4 × 4 to 26 × 26 cm^2^. As with 3D dose reconstruction within the phantom, the patient dose was reconstructed via a similar approach. During this process, two main differences should be considered: anatomical structures are inhomogeneous along the ray line but feature different densities. It is impossible to account for the change in scattered radiation as the ray line passes through structures with different densities using the inhomogeneity correction algorithm, therefore, there is a limitation to the dose calculation accuracy in the presence of relevant inhomogeneity^31^. Additionally, the dose is empirically calculated based on measured data, which is characterized by ignoring different penumbra sizes of ray heterogeneity^20^. During optimization, Oncentra MasterPlan relies on a simplified value decomposition algorithm for fast pencil beam dose calculation. For final dose calculation, the user can choose between pencil beam convolution (PBC) and CCC algorithms^37,38^, in which CCC includes more accurate inhomogeneity corrections^39^. The CCC is a superposition method based on a point kernel convolution with a fixed number of different directions along which the energy is transported from each grid point in the patient^40^. Strikingly, CCC offers superior dose delivery in all clinically relevant cases, which is still relatively close to Monte Carlo, even in the extreme case of ρ_lung_ = 0.01 g/cm^3,41^. Our findings showed that the Pdd/% averages of the abdomen/gastric and pelvic sites were relatively smaller, while the Pdd/% averages of the mediastinum and lung were somewhat larger. Specifically, Compass reconstructed doses using the CCC algorithm the same as TPS, making Pdd% values more indicative of anatomical locations and mass density on reconstruction accuracy. Although Eclipse TPS and Verisoft software use different types of algorithms, in regard to small tissue inhomogeneity, such as the abdomen and pelvis, the DVHs calculated by the two algorithms show a slightly smaller difference. A similar report showed that the dose differences were greater for treatment sites with relevant inhomogeneity, such as the lungs^20^. Given the relationship between Hounsfield unit values and different tissue densities of a CT scanner used in AAA, for mass densities ranging from 0 to 3.0 g/cm^3^, Eclipse defines six biological tissue types: air, lung tissue, adipose tissue, muscle, cartilage, and bone^42,43^. One would expect significant variations in Pdd/% values between thorax and abdomen/pelvic sites irradiating various low- and high-density structures.

To obtain accurate numerical estimates, linear interpolation should satisfy the premise that numerical changes in relevant physical parameters within known data point regions are linear^13^. We hypothesize that the dose distribution generated by the linac in the dosimetric phantom of the ion chamber detector is a two-dimensional continuous signal distribution, and non-interpolation measurement results are equivalent to sampling this continuous distribution. According to the Nyquist sampling theorem, to ensure that the sampled signal truly retains the original signal information, the sampling frequency must be at least twice the highest frequency in the original signal^44^. Therefore, if the measured dose distributions vary between two independent detectors in the ion chamber array, it is not possible to reconstruct the actual dose distribution based on the measurement results of the detector array^12^. When applied to the dose reconstruction on human anatomical structures, reconstruction accuracy is better for anatomical sites with a smaller mass density range and tissue inhomogeneity.

Due to better approximation of dose change, the cubic spline interpolation algorithm can lead to a superior γ pass rate, superior to that of linear interpolation, which basically reflects nonlinear variation in dose distributions between intrinsic intervals of the ion chamber detector array^12,45^. In addition, since dose variations between two sampling points are nonlinear, the bicubic interpolation algorithm can better approximate dose distributions between two sampling points than the linear interpolation algorithm^45^. Previous research used spline interpolation, linear interpolation, and cubic interpolation methods to determine whether different interpolations may obtain significant differences in the γ pass rate and absolute γ values^15^. They found that spline algorithms provided the best agreement between non-interpolated and interpolated data, namely, the highest γ pass rate and smallest absolute γ values. In comparison, linear interpolation produces the lowest γ pass rate and largest absolute γ values. A large dose gradient region may exist between the tumor target volume and OARs in VMAT dose distributions, while stereotactic radiotherapy requires a steeper dose drop. In high-dose gradient regions, dose distribution at the gradient edge cannot be shown truly due to smoothing and blurring effects of the linear interpolation algorithm^46–49^. Taken together, these results reveal that the accuracy of the linear interpolation is slightly poor. Compared with parabolic interpolation and other interpolation methods, the advantages of linear interpolation are simple and convenient^13^. Moreover, linear interpolation only uses the values of two points to estimate the missing values between them, at least in part, these interpolated values are often affected by various accidental factors, such as dose rate. In rotational gantries, VMAT dose rates change constantly, and the points with low dose response may be interpolated as no response when the dose rate is low (˂ 5 cGy/min)^50^. Accordingly, Verisoft and Compass software may suffer from error interpolation on the detector measurement signal at low dose rates. Of note, in individual control point planar dosimetry, the dose in the treatment field is measured after a single delivery, but the challenge is interpolating the measured dose accurately. In deconstructing a VMAT plan into segments, a high dose, low gradient volume often turns into a variety of very complex dose patterns.

In conclusion, our study provides insights into linear interpolating new dose points in the VMAT dosimetry using ion chamber detector arrays. Interpolation errors can be reduced by increasing the number of sampling points and decreasing the spacing between adjacent detectors. Anatomical structures with a smaller mass density range can lead to better volumetric dose reconstruction, which may be explained by the fact that a linear numerical change in dose distribution within known data point regions can contribute to an accurate numerical estimate using the linear interpolation algorithm. However, dose variations between two sampling points are nonlinear, therefore, alternative data interpolation methods are needed to more effectively assess the clinical significance of radiotherapy planning quality assurance results.

## Data Availability

Figures 7, 8, 9 and 10 and Table 2 are provided as source data files. Other datasets used and/or analysed during the current study are available from the corresponding author on reasonable request.

## References

[1] Sun WJ (2021). Optimization of collimator angles in dual-arc volumetric modulated arc therapy planning for whole-brain radiotherapy with hippocampus and inner ear Sparing. Sci. Rep..

[2] Zhand HY (2022). Assessment of statistical process control based DVH action levels for systematic multi-leaf collimator errors in cervical cancer RapidArc plans. Front. Oncol..

[3] Sayah R (2020). Dosimetric impact of switching from AAA to Acuros dose-to-water and dose-to-medium for RapidArc plans of nasopharyngeal carcinomas. Cancer Radiother..

[4] National Cancer Center/ National Cancer Quality Control Center (2020). Practice guideline of patient-specific dosimetric verification for intensity-modulated radiotherapy. Chin. J. Radiat. Oncol..

[5] Petrucci E (2021). Delta Discover transmission detector: A comprehensive characterization for in-vivo VMAT monitoring. Phys. Med..

[6] Hunter M (2020). Survey results of 3D-CRT and IMRT quality assurance practice. J. Appl. Clin. Med. Phys..

[7] Steers JM, Fraass BA (2021). IMRT QA and gamma comparisons: The impact of detector geometry, spatial sampling, and delivery technique on gamma comparison sensitivity. Med. Phys..

[8] Sathiyan S, Ravikumar M, Varatharaj C, Sanjay SS (2010). Dosimetric study of 2D ion chamber array matrix for the modern radiotherapy treatment verification. J. Appl. Clin. Med. Phys..

[9] Nelms BE (2012). VMAT QA: Measurement-guided 4D dose reconstruction on a patient. Med. Phys..

[10] Bruschi A, Esposito M, Pini S (2018). How the detector resolution affects the clinical significance of SBRT pretreatment quality assurance results. Phys. Med..

[11] Guo YX (2021). Analysis on dosimetric verification of flattening filter free conventional segmentation partial arc RapidArc plans. J. Radiat. Res. Radiat. Process..

[12] Zhang JY (2010). The effort to gamma pass rates of interpolation during the 2D chamber array IMRT QA. Chin. J. Med. Phys..

[13] Ma DS, Dong N (2015). Numerical Computation Method.

[14] Bäck A (2015). Quasi 3D dosimetry (EPID, conventional 2D/3D detector matrices). J. Phys. Conf. Ser..

[15] Hussein M, Clark CH, Nisbet A (2017). Challenges in calculation of the gamma index in radiotherapy–towards good practice. Phys. Med..

[16] Huang JY (2014). Effects of spatial resolution and noise on gamma analysis for IMRT QA. J. Appl. Clin. Med. Phys..

[17] Dorenlot A (2013). Retrospective of 300 delivery quality assurance of patients treated with the tomotherapy HI-ART 2 using the IBA matrixx-evolution. Phys. Med..

[18] Zhang XL, Yang RJ, Li J (2019). Analysis of dosimetric verification results of stereotactic body radiotherapy. Chin. J. Radiol. Med. Prot..

[19] Bipasha P (2021). Comparative performance analysis of 2D and 3D gamma metrics for patient specific QA in VMAT using Octavius 4D with 2D-Array 1500. Phys. Med..

[20] Song JY, Ahn SJ (2018). Dosimetric evaluation of the compass program for patient dose analysis in IMRT delivery quality assurance. PLoS ONE.

[21] Ramesh B (2011). Patient-specific 3D pretreatment and potential 3D online dose verification of Monte Carlo calculated IMRT prostate treatment plans. Int. J. Radiat. Oncol. Biol. Phys..

[22] Gregoire V (2018). Delineation of the primary tumour Clinical Target Volumes (CTV-P) in laryngeal, hypopharyngeal, oropharyngeal and oral cavity squamous cell carcinoma: AIRO, CACA, DAHANCA, EORTC, GEORCC, GORTEC, HKNPCSG, HNCIG, IAG-KHT, LPRHHT, NCIC CTG, NCRI, NRG Oncology, PHNS, SBRT, SOMERA, SRO, SSHNO, TROG consensus guidelines. Radiother. Oncol..

[23] Chapet O (2005). CT-based definition of thoracic lymph node stations: An atlas from the University of Michigan. Int. J. Radiat. Oncol..

[24] Lim K (2011). Consensus guidelines for delineation of clinical target volume for intensity-modulated pelvic radiotherapy for the definitive treatment of cervix cancer. Int. J. Radiat. Oncol..

[25] Roels S (2006). Definition and delineation of the clinical target volume for rectal cancer. Int. J. Radiat. Oncol..

[26] Chen J (2022). Evaluation of auto-planning inVMAT for locally advanced nasopharyngeal carcinoma. Sci. Rep..

[27] Cordoba A (2015). Safety of adjuvant intensity-modulated postoperative radiation therapy in endometrial cancer: Clinical data and dosimetric parameters according to the International Commission on Radiation Units (ICRU) 83 report. Rep. Pract. Oncol. Radiother..

[28] Sun Y (2013). Which T category of nasopharyngeal carcinoma may benefit most from volumetric modulated arc therapy compared with step and shoot intensity modulated radiation therapy. PLoS ONE.

[29] Junichi F (2022). Multi-institution model (big model) versus single-institution model of knowledge-based volumetric modulated arc therapy (VMAT) planning for prostate cancer. Sci. Rep..

[30] Chen S (2020). High-throughput in situ root image segmentation based on the improved DeepLabv3+ method. Front. Plant Sci..

[31] Thomas SJ (1999). Relative electron density calibration of CT scanners for radiotherapy treatment planning. Br. J. Radiol..

[32] Rosenbloom ME (1985). Central axis depth dose data for use in radiotherapy. Br. J. Radiol..

[33] Van EA (2007). On-line quality assurance of rotational radiotherapy treatment delivery by means of a 2D ion chamber array and the Octavius phantom. Med. Phys..

[34] Knöös T (2006). Comparison of dose calculation algorithms for treatment planning in external photon beam therapy for clinical situations. Phys. Med. Biol..

[35] Ojala JJ (2014). Performance of dose calculation algorithms from three generations in lung SBRT: Comparison with full Monte Carlo-based dose distributions. J. Appl. Clin. Med. Phys..

[36] Halvorsen PH, Hariharan N, Morelli ZT, Iftimia IN (2021). Modeling of kyphoplasty cement for accurate dose calculations. J. Appl. Clin. Med. Phys..

[37] Zhao Y (2015). A clinical study of lung cancer dose calculation accuracy with Monte Carlo simulation. Radiat. Oncol..

[38] Delana A, Barbareschi A, Consorti R, Falco MD (2020). Dose calculation accuracy in proximity of a pacemaker: A multicenter study with threecommercial treatment planning systems. Phys. Med..

[39] Ahnesjö A, Aspradakis MM (1999). Dose calculations for external photon beams in radiotherapy. Phys. Med. Biol..

[40] Aarup LR (2009). The effect of different lung densities on the accuracy of various radiotherapy dose calculation methods: Implications for tumour coverage. Radiother. Oncol..

[41] Nisbet A (2004). Dosimetric verification of a commercial collapsed cone algorithm in simulated clinical situations. Radiother. Oncol..

[42] Kan MW, Leung LH, So RW, Yu PK (2013). Experimental verification of the Acuros XB and AAA dose calculation adjacent to heterogeneous media for IMRT and RapidArc of nasopharygeal carcinoma. Med. Phys..

[43] Han T, Mikell JK, Salehpour M, Mourtada F (2011). Dosimetric comparison of Acuros XB deterministic radiation transport method with Monte Carlo and model-based convolution methods in heterogeneous media. Med. Phys..

[44] Yu FH (2022). Data reduction in phase-sensitive OTDR with ultra-low sampling resolution and undersampling techniques. Sensors (Basel).

[45] Cheng YX, Xiu Y, Li MH, Hu YM (2015). Converting low density 2D array dose distributions to high density 2D array ones. Chin. J. Med. Phys..

[46] Qi Y (2013). A study of dose verification method in the IMRT. China Cancer.

[47] Wuu CS, Xu Y (2006). Three-dimensional dose verification for intensity modulated radiation therapy using optical CT based polymer gel dosimetry. Med. Phys..

[48] Nasonov AV, Krylov AS (2011). Finding areas of typical artifacts of image enhancement methods. Pattern Recognit. Image Anal..

[49] Hsia SC, Chen MH, Tsai PS (2006). VLSI implementation of low-power high-quality color interpolation processor for CCD camera. IEEE Trans. Very Large Scale Integr. Syst..

[50] Lu J, Hu CR, Cai YG, Yin XJ, Hu JQ (2013). Application of three dimensional dose verification system in volume rotation intensification. Chin. J. Radiol. Med. Prot..

